# Functional and bioactive properties of *Larimichthys polyactis* protein hydrolysates as influenced by plasma functionalized water-ultrasound hybrid treatments and enzyme types

**DOI:** 10.1016/j.ultsonch.2022.106023

**Published:** 2022-05-04

**Authors:** Okon Johnson Esua, Da-Wen Sun, Jun-Hu Cheng, Huifen Wang, Mingchun Lv

**Affiliations:** a School of Food Science and Engineering, South China University of Technology, Guangzhou 510641, China; b Academy of Contemporary Food Engineering, South China University of Technology, Guangzhou Higher Education Mega Center, Guangzhou 510006, China; c Engineering and Technological Research Centre of Guangdong Province on Intelligent Sensing and Process Control of Cold Chain Foods, & Guangdong Province Engineering Laboratory for Intelligent Cold Chain Logistics Equipment for Agricultural Products, Guangzhou Higher Education Mega Centre, Guangzhou 510006, China; d Food Refrigeration and Computerized Food Technology (FRCFT), Agriculture and Food Science Centre, University College Dublin, National University of Ireland, Belfield, Dublin 4, Ireland

**Keywords:** Alcalase, Papain, Chymotrypsin, Hydrolysis, Cavitation, Reactive oxygen species

## Abstract

The effects of plasma functionalized water (PFW) and its combination with ultrasound (UPFW) on the functional and bioactive properties of small yellow croaker protein hydrolysates (SYPHs) produced from three enzymes were investigated. Fluorescence and UV–Vis spectroscopy indicated that SYPHs tended to unfold with increasing intensity and shift in wavelengths to more flexible conformations under PFW and UPFW treatments. Particle size distribution and microstructure analysis revealed that treatments could disrupt aggregation of protein molecules to increase the roughness, specific surface area, and decrease the particle size of peptides during hydrolysis. The partially denatured structure of SYPHs induced by treatments increased the susceptibility of the fish proteins to exogenous enzymes, thereby accelerating the hydrolytic process to yield peptides with improved solubility, decreased emulsifying and foaming properties, and improved enzyme-specific antioxidant properties. The results revealed that the functionality of SYPHs was influenced by the treatment method and the enzyme type employed.

## Highlights

• Treatment induced conformational changes and structural unfolding of peptide chains.
• Peptides with increased roughness, surface area and smaller particles were created.
• Accelerated hydrolysis produced soluble peptides with improved bioactive properties.
• Emulsifying and foaming properties were reduced.
• Hydrolysates Functionality was affected by processing conditions and enzyme type.

## 1 Introduction

Biologically active peptides from fish proteins have attracted increasing attention in recent times as their bioactivity makes them suitable ingredients for functional food development [1]. Fish protein hydrolysates are involved in oxidative stress prevention with anticancer, antimicrobial, anti-inflammatory, antidiabetic, antihypertensive, and anticholesterolemic properties, and can be prepared through enzymatic, chemical and sub-critical hydrolysis [2], [3], [4]. Enzymatic hydrolysis is commonly used due to the safety, efficiency and characteristic selectivity of enzymes to substrates, thus, investigating the enzyme-specific susceptibility of fish muscle proteins to hydrolysis is important since they influence the bioactivity of the hydrolysates produced.

Food processing can modify protein structure for improving sensitivity to enzymolysis and influencing bioactive properties. For example, ultrasound and microwave treatments disrupted protein aggregation to produce smaller particles that improved the susceptibility of golden threadfin bream myofibrillar proteins (MP) to exogenous enzymes, thereby expediting hydrolysis to yield high peptide concentration with improved bioactivity [5]. In other studies, pressure-assisted enzymatic hydrolysis of tilapia by-products produced hydrolysates with improved soluble protein and antioxidant activities, while ultrasound treatment, high-pressure homogenization, and spray drying improved the antihypertensive potential, angiotensin-converting enzyme inhibitory and antioxidant activities of sardine hydrolysates [1], [6]. Therefore, it is necessary to evaluate the impact of food treatments on the functional and bioactive properties of fish protein hydrolysates.

Plasma functionalize water (PFW) is a developing broad-spectrum sanitiser characterized by reduced pH from exposing water to electrons and ionized compounds at the cold plasma-liquid interface that affects the equilibria of several reactive oxygen and nitrogen species (RONS) like H_2_O_2_, O_2_^–^, •OH, O_3_, ONOO^–^, NO_3_^–^ and NO_2_^–^ [7], [8], [9], [10], [11]. Some of the species like O_2_^–^ and OH^–^ have relatively short half-lives, while species like NO_2_^–^, NO_3_^–^, O_3_ and H_2_O_2_ are long-term half-life species [10], [12], [13]. These reactive chemistries, together with the protonated peroxunitrous acid (ONOOH) formed from the reaction with superoxide anion radicals are responsible for the oxidation of proteins, lipids, DNA, and RNA. Previous studies suggested that cold plasma could modify protein conformation with better physicochemical properties by affecting hydrogen bonds and exposing hydrophobic groups [14]. On the other hand, ultrasound treatment (UST) is characterized by cavitation effects of strong shock waves accompanied by localized high temperature, pressure and pyrolysis reactions that can also generate RONS, and its successful combination with PFW for further improving the safety and quality of fish have been reported [15], [16], [17], [18], [19].

In addition, UST has also been successfully combined with nonthermal techniques for further improving protein structure and enhancing functionality. For instance, the combination of 300 W UST and 100 W microwave treatment was an optimal method for disrupting highly-ordered aggregates of MP from golden threadfin bream into smaller fragments and generating peptides with improved functionality, when compared with UST or microwave treatments alone [5]. Appropriate UST power during UST-assisted freezing was reported to promote the formation of more uniform and smaller ice crystals for minimizing the changes in protein structure and improving the thermal stability of common carp proteins [20]. Also, UST-assisted grafting of dextran conjugates to MP induced Maillard reactions that led to the loss of ordered secondary structures and improvements in emulsifying ability and stability, while UST-assisted covalent reactions of epicatechin gallate and chicken MP ensured more unfolding of protein structure with improved bioactivity [20], [21].

Despite the promising potential of PFW and its combination with UST for enhancing the safety and quality of seafood products, their effects on seafood protein hydrolysates, which is a significant feature of the process appraisal, have not been investigated. Small yellow croaker (*Larimichthys polyactis*) is an important economic resource and essential commodity in Western Pacific countries especially in China due to its deliciousness and high nutritional content, but overexploitation has heavily reduced its biomass [23], [24]. With the successful development of new management strategies of artificial breeding for the sustainable utilization of this species [23], it is necessary to study the effects of developing seafood decontamination treatments on its protein hydrolysate.

Consequently, the current study was designed to evaluate the changes in the functional and bioactive properties of small yellow croaker protein hydrolysates (SYPHs) derived from three different enzymes during treatment with PFW and its combination with UST. Spectral measurements, particle size distribution (PSD) and atomic force microscopy (AFM) were also evaluated for further obtaining information on the conformational and morphological changes.

## 2 Materials and methods

### 2.1 Enzymes and reagents

Papain was supplied by Yuanye Biotechnology Co., Ltd. (Shanghai, China), hydrogen peroxide (H_2_O_2_) was purchased from Chengdu Chron Chemicals Co., Ltd. (Chengdu, China) and Tris-HCl buffer was supplied by Nobleryder Technology Co. Ltd. (Beijing, China). Alcalase© 2.4 L, Chymotrypsin, potassium ferricyanide (C_6_FeK_3_N_6_), ferrozine (C_20_H_12_N_4_Na_2_O_6_S_2_), sodium dodecyl sulphate (SDS), iron (II) chloride (FeCl_2_), ferrous sulphate (FeSO_4_), 1,10-phenanthroline (C_12_H_8_N_2_), sodium hydrogen phosphate (Na_2_HPO_4_), sodium dihydrogen phosphate (NaH_2_PO_4_), trichloroacetic acid (C_2_HCl_3_O_2_) and methyl red (C_15_H_15_N_3_O_2_) were acquired from Aladdin Industrial Co. (Shanghai, China). Hydrochloric acid (HCl) was obtained from Guangzhou Rongman Technology Co., Ltd. (Guangzhou, China), ethanol (CH_3_CH_2_OH) was purchased from Tianjin Fuyu Fine Chemical Co., Ltd. (Tianjin, China), bromocresol green (C_21_H_14_Br_4_O_5_S), iron (III) chloride (FeCl_3_) and 1,1-diphenyl-2-picrylhydrazyl (DPPH) were bought from Shanghai Macklin Biochemical Co., Ltd. (Shanghai, China). Sodium hydroxide (NaOH) and boric acid (H_3_BO_3_) were procured from Sinopharm Chemical Reagent Co., Ltd. (Shanghai, China).

### 2.2 Sample preparation and PFW generation

**Fig. 1:**
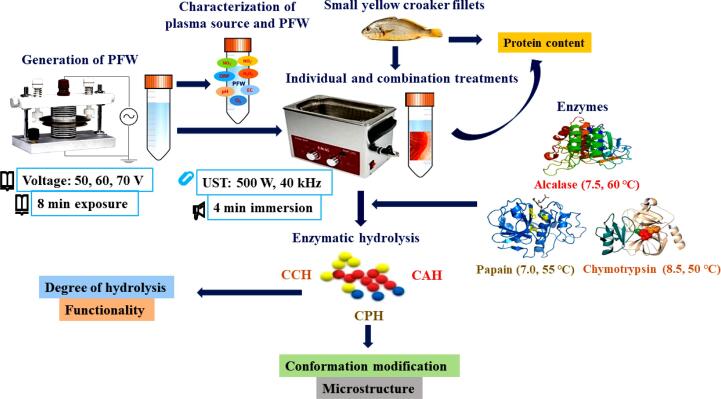
Schematic representation of experimental procedure.

A schematic representation of the experimental procedure for the current study is presented in Fig. 1. Small yellow croaker (length: 15 – 19 cm and weight: 45.24 – 58.66 g) was purchased from Shanwei Cathay Food Industries Ltd., Shanwei, China, and transported in an icebox to the research laboratory of the Academy of Contemporary Food Engineering, South China University of Technology, Guangzhou, China. Experimental samples of 20 g were obtained after evisceration and filleting and stored at − 20 °C pending treatment. PFW was generated from a dielectric barrier discharge (DBD) system (CTP-2000 K, Nanjing Suman Electronics Co., Ltd., Nanjing, China) described in previous studies [10], [25]. An amount of 20 mL deionized water (DW) contained in a petri dish was placed between the low-voltage and high-voltage electrodes and exposed to plasma discharge at a distance of 5 mm from the water surface and input voltage of 50, 60, and 70 V for 8 min. The PFW generated were designated as PFW50, PFW60, and PFW70, and allowed a cooling time of 2 min to void the temperature effect.

### 2.3 Optical emission spectroscopy and characterization of PFW

The RONS produced by the DBD plasma during the generation of PFW were identified by optical emission spectroscopy (OES) using a computer-controlled spectrometer (HR2000 +, Ocean Optics Inc., FL, USA). The spectrometer was fitted with 0.66 mm resolution fibre optic and operated in the visible and ultraviolet spectral region of 200 – 1100 nm. A distance of 5 mm was maintained between the plasma discharge and the probe, and the emission lines of various reactive species were identified from comparison with the atomic spectra database of the National Institute of Standards and Technology [26]. For characterizing the prepared PFW, the temperature, pH, oxidation–reduction potential (ORP), electrical conductivity (ECT), hydrogen peroxide (H_2_O_2_) and ozone (O_3_) contents were measured as described in previous studies [10].

### 2.4 Treatments and protein analysis

Individual treatment with PFW was achieved by immersing the samples in 40 mL of each of PFW50, PFW60, and PFW70 contained in a beaker and placed on an orbital shaker (WSZ-10A, Shanghai Yiheng Technology Co., Ltd., Shanghai, China) for 4 min at 150 rpm and room temperature of 25 ℃. The treated samples were designated as P50S, P60S, and P70S, respectively. For UST, samples were immersed in 40 mL DW and treated at room temperature of 25 ℃ for 4 min in an ultrasound bath (SB25-12D, Ningbo Xinzhi Ultrasonic Equipment Co., Ltd., Ningbo, China) with internal dimensions of 500 x 300 x 150 mm (L x W x H). The ultrasound bath was operated at a frequency and power of 40 kHz, 500 W, respectively, and the treated samples were designated as UDS. Ultrasound energy is typically lost as heat during irradiation in a liquid medium. Thus, the calorimetric method described in our earlier study [18] was employed for estimating the effective acoustic intensity of 12.21 W/L following immersion depth of 80 mm from the bottom of the tank and final temperature of 25.7 ℃, using the equation below:

(1) $$P_{\mathrm{diss}}=mC_{p}\left(\frac{\mathrm{dT}}{\mathrm{dt}}\right)$$

(2) $$Acoustic\;intensity\;=\frac{P_{\mathrm{diss}}}{V}$$

where $P_{\mathrm{diss}}$ is ultrasonic power dissipated into the water in the tank, m is the mass of the water in the tank (kg), $C_{p}$ is the specific heat capacity of water (4187 J/kg K), (dT/dt) is the slope of the temperature versus time curve for the treatment time of 4 min, and V is the volume of water in the tank (L).

For the combination treatments, samples were first immersed in PFW and subjected to UST as described above, and designated as UP50S, UP60S and UP70S, respectively. For the control, samples were immersed in 40 mL DW for 4 min, and these samples were designated as DWS. Protein analysis of control and treated samples were performed according to the Kjeldahl method [27], and the total crude protein was determined using a conversion factor of 6.25.

### 2.5 Preparation of SYPHs

The SYPHs were prepared according to the methods of Khantaphant et al. [28] and Lima et al. [29] with modifications. The treated samples (UDS, P50S, P60S, P70S, UP50S, UP60S, and UP70S) and the control (DWS) were homogenized (IKA T18 Digital ULTRA-TURRAX, IKA Equipment Co., Germany) in 50 mL DW at 80 ℃ for 10 min to inactivate endogenous enzymes. Thereafter, the homogenous mixtures were separately equilibrated to the desired pH for the different enzyme activity with 50 mM Tris-HCl buffer (Alcalase: 7.5, Chymotrypsin: 8.5, Papain: 7.0), and the details of the enzymes are presented in Table 1a. Hydrolysis was initiated by adding 2% of the different enzymes (w/w) to the respective equilibrated mixtures and monitored in a shaking water bath (HH-501, Changzhou Aohua Instrument Co., Ltd, Changzhou, China) at 60, 50 and 55 ℃, respectively, for Alcalase, Chymotrypsin and Papain. The reaction was terminated after 2 h by heating the mixture for 10 min at 90 ℃, followed by centrifuging (JW-3024HR, Anhui Jiaven Equipment Industry Co., Ltd., Hefei, China) at 8960 × *g*, 4 ℃ for 10 min. The supernatants were designated as SYPHs prepared from Alcalase (CAH), Chymotrypsin (CCH) and Papain (CPH), and a total of 72 hydrolysate samples were obtained and stored at –80 ℃ until further analysis.

**Table 1:** Enzyme properties and physicochemical characteristics of PFW.

**a**	**Enzyme**	**Enzyme commission number**		**Type**	**Source**	**Temperature range (℃)**		**pH range**	**Activity**
	Alcalase	3.4.21.62		Serine protease	*Bacillus licheniformis*	50 – 70		6.5 – 8.5	2.4 AU/g
	Chymotrypsin	3.4.21.1		Serine protease	Bovine pancreas	40 – 60		7.0 – 9.0	45 U/mg
	Papain	3.4.22.2		Cysteine protease	*Carica papaya*	40 – 70		5.0 – 8.0	100 TU/mg
**b**	**Liquids**	**Temperature (℃)**		**H_2_O_2_ (µM)**	**O_3_ (mg/L)**	**ECT (mS/cm)**		**pH**	**ORP (mV)**
	DW	25.30 ± 0.40^b^		0.0024 ± 0.001^c^	0.07 ± 0.01^b^	0.0094 ± 0.0013^b^		6.67 ± 0.06^a^	304.25 ± 18.45^b^
	PFW50	45.90 ± 1.40^a^		1.3009 ± 0.128^b^	3.19 ± 0.02^a^	2.0800 ± 0.0750^a^		2.88 ± 0.02^b^	575.53 ± 4.33^a^
	PFW60	47.40 ± 1.30^a^		2.0067 ± 0.111^a^	3.71 ± 0.09^a^	2.5475 ± 0.1075^a^		2.71 ± 0.01^bc^	583.92 ± 4.88^a^
	PFW70	49.80 ± 1.20^a^		2.0771 ± 0.010^a^	5.05 ± 0.67^c^	2.7275 ± 0.1925^a^		2.60 ± 0.03^c^	598.42 ± 0.98^a^
**c**	**Protein content (g/kg)**	**DWS**	**UDS**	**P50S**	**P60S**	**P70S**	**UP50S**	**UP60S**	**UP70S**
		204.27 ± 8.40^A^	188.19 ± 5.11^B^	180.15 ± 6.10^BC^	176.93 ± 9.48^BC^	172.11 ± 3.24^CD^	160.85 ± 7.60^DE^	151.20 ± 5.89^EF^	141.54 ± 9.09^F^

AU: Anson unit, the amount of enzyme that releases 1 meq of tyrosine from urea-denatured haemoglobin per minute at pH 7.5 and 25℃; U: unit of protease activity, the amount of enzyme that will hydrolyse 1.0 µmol of Benzoyl-L-tyrosine ethyl ester per minute at pH 7.8 and 25℃; TU: tyrosine unit, the amount of enzyme that releases 1 mg equivalent of tyrosine per minute from a specified substrate under the conditions of the hydrolysis. ORP: oxidation–reduction potential; ECT: electrical conductivity. DWS: immersed in deionized water; UDS: ultrasound treatment; P50S: immersed in PFW50; P60S: immersed in PFW60; P70S: immersed in PFW70; UP50S; immersed in PFW50 and treated with ultrasound; UP60S: immersed in PFW60 and treated with ultrasound; UP70S: immersed in PFW70 and treated with ultrasound. Values are mean ± standard error of measurements (*n* = 3); different lowercase letters in the same column and uppercase letters in the same row are significantly different (*p* < 0.05).

### 2.6 Intrinsic fluorescence spectroscopy

Intrinsic fluorescence spectra of SYPHs were acquired in the wavelength range of 300 – 600 nm and excitation wavelength of 280 nm at a slit width of 3 nm from a fluorescence spectrophotometer (RF-6000, Shimadzu Co., Kyoto, Japan), for observing the structural changes [14]. Measurements were done in triplicates against the buffer used for hydrolysis as blank.

### 2.7 UV–Vis absorption spectroscopy

The UV–Vis spectra of SYPHs were acquired within the wavelength range of 220 – 330 nm at the rate of 10 nm/min from a UV–Vis-NIR spectrophotometer (UV-1800, Shimadzu Co., Kyoto, Japan), for elucidating their conformational changes [1]. Triplicate measurements were carried out for ensuring reproducibility against the buffer used for hydrolysis as blank.

### 2.8 Particle size distribution (PSD) and particle size evaluation

Particle size distribution (PSD) of SYPHs were evaluated by a laser particle sizer (Mastersizer 2000, Malvern Instruments Ltd., Worcestershire, UK). Hydrolysates were dispersed in DW at refractive and uniformity indices of 1.330 and 2.15, respectively, and the specific surface area (SSA) and volume-weighted mean diameter (D_43_) were obtained from the Malvern standard operation procedure software (Malvern Instruments Ltd., Worcestershire, UK).

### 2.9 Atomic force microscopy (AFM)

The microstructure and surface roughness of SYPHs were investigated via AFM (SmartSPM-1000, HORIBA France SAS, Lille, France). Hydrolysates were diluted to 40 µg/mL and 10 µL of the diluted solution were dropped on fresh mica surface, air-dried in a fume hood for 1 h, scanned at a rate of 1.0 Hz and step of 2 µm in the tapping mode, and the images were analyzed from the AIST-NT SPM control version 3.5.133 software (AIST-NT Inc., California, USA).

### 2.10 Degree of hydrolysis (DoH)

The DoH of SYPHs was evaluated by the ratio of 10% trichloroacetic acid (TCA)-soluble nitrogen in hydrolysates to the total nitrogen in fish samples as documented by Altınelataman et al. [30] with modifications. Aliquots of 10 mL were removed at intervals of 0, 30, 60, 90, and 120 min during hydrolysis and mixed with 10 mL 20% TCA followed by centrifuging at 10, 000 rpm and 4 ℃ for 10 min to create 10% TCA-soluble fractions. Nitrogen contents of the hydrolysates were measured by the Kjeldahl method [27], and the DoH was determined from the equation below:

(3) $$\mathrm{DoH}\;\left(\%\right)=\frac{10\%TCA-soluble\;nitrogen\;in\;hydrolysates}{\mathrm{Total}\;\mathrm{nitrogen}\;\mathrm{in}\;\mathrm{fish}\;\mathrm{sample}}*100$$

### 2.11 Functional properties of SYPHs

#### 2.11.1 Protein solubility and turbidity

The protein solubility of SYPHs was expressed as the percentage of the dissolved protein in the hydrolysates after centrifugation to the protein content before centrifugation as shown below:

(4) $$\mathrm{Protein}\;\mathrm{solubility}\left(\%\right)=\frac{\mathrm{Protein}\;\mathrm{content}\;\mathrm{after}\;\mathrm{centrifugation}}{\mathrm{Protein}\;\mathrm{content}\;\mathrm{before}\;\mathrm{centrifugation}}*100$$

The protein contents of hydrolysates were determined before and after centrifugation from the calibration curve of standard bovine serum albumin using the Bradford protein assay kit (Beyotime Biotechnology, Shanghai, China) and by measuring the absorbance at 595 nm from a multi-mode microplate reader (SpectraMax i3, Molecular devices, Shanghai, China).

For obtaining the turbidity, the method of Ekezie et al. [14] was employed by measuring the absorbance at 350 nm from the multi-mode microplate reader and the value of the absorbance was expressed as the turbidity of SYPHs.

#### 2.11.2 Emulsifying properties

The emulsifying properties were evaluated as described by Hemker et al. [1] with modifications, by homogenizing a mixture of 10 mL SYPHs and 4 mL soybean oil for 2 min at 10,000 rpm. Thereafter, 1.5 mL of 0.1% SDS solution was thoroughly mixed with 50 µL of the mixture taken from the bottom at 0 and 10 min after homogenization and the absorbance was measured at 500 nm from the multi-mode microplate reader. The emulsifying activity index (EAI) and emulsifying stability index (ESI) were calculated from the equations below:

(5) $$\mathrm{EAI}\;(m^{2}/g)=\frac{2*2.303A_{o}}{0.25*w}$$

(6) $$ESI\;\left(min\right)=\frac{A_{o}}{A_{o}-A_{10}}\Delta T$$

where $A_{o}$ and $A_{10}$ are the absorbance of the sample at 0 and 10 min, respectively, ΔT is the change in time and w is the weight of protein (g).

#### 2.11.3 Foaming properties

Foaming properties were determined as described by Ekezie et al. [14] with modifications, by homogenizing 10 mL of SYPHs at 10,000 rpm for 2 min in a graduated cylinder for incorporating air into the solution. The whipped-protein solution was allowed to stand for 30 min at room temperature of 25 ℃ and the volume was recorded. The foaming capacity (FCY) and foaming stability (FSY) were obtained from the equations below:

(7) $$FCY\left(\%\right)=\frac{V_{w}}{V_{i}}*100$$

(8) $$FSY\left(\%\right)=\frac{V_{30}}{V_{i}}*100$$

where $V_{i}$, $V_{w}$, and $V_{30}$ are initial volume of protein solution, volume immediately after whipping, and volume after 30 min, respectively.

### 2.12 Antioxidant activities of SYPHs

#### 2.12.1 2,2-Diphenyl-1-picrylhydrazyl (DPPH) radical scavenging activity assay

The scavenging effect of SYPHs on the DPPH free radical was measured according to the method of Li et al. [5] with modifications. Briefly, 2.0 mL of 0.2 mM DPPH solution dissolved in 95% CH_3_CH_2_OH was mixed with 2.0 mL of SYPHs and kept in the dark for 30 min at room temperature of 25 ℃. Afterwards, the absorbance was measured from the multi-mode microplate reader at 517 nm and the scavenging activity was calculated using the following equation:

(9) $$DPPH\;radical\;scavenging\;activity\;\left(\%\right)=\left(\frac{A_{s}-A_{b}}{A_{s}}\right)*100$$

where $A_{s}$ is the absorbance of protein hydrolysates, $A_{b}$ is the absorbance of the blank (mixture with ethanol replacing protein hydrolysates).

#### 2.12.2 Hydroxyl radical scavenging activity assay

The hydroxyl radical scavenging activity was determined from the method of Zou et al. [31] with modifications. Briefly, 600 µL of 5 mM FeSO_4_ was mixed with 600 µL of 5 mM C_12_H_8_N_2_ and 400 µL of 0.2 M sodium phosphate buffer (pH 7.4). Subsequently, 600 µL of SYPHs and 800 µL of 0.01% H_2_O_2_ were added to the mixture and incubated for 60 min at 37 ℃. The absorbance of the mixture was measured at 536 nm from the multi-mode microplate reader and the hydroxyl radical scavenging activity was obtained as presented below:

(10) $$Hydroxyl\;radical\;scavenging\;activity\;\left(\%\right)=\left(1-\frac{A_{s}}{A_{b}}\right)*100$$

where $A_{s}$ is the absorbance of the sample, $A_{b}$ is the absorbance of the blank solution without hydrolysates.

#### 2.12.3 Metal-chelating activity

The pro-oxidative Cu^2+^ chelating ability was determined from the method of Zou et al. [32] by mixing 1 mL of SYPHs with 0.05 mL of 2 mM FeCl_2_ and 2 mL DW. Thereafter, 0.1 mL of 5 mM C_20_H_12_N_4_Na_2_O_6_S_2_ was added, mixed thoroughly and incubated for 20 min at room temperature of 25 ℃. The absorbance of the solution was read at 562 nm from the multi-mode microplate reader and the metal-chelating activity was calculated as expressed below:

(11) $$\mathrm{Metal}-chelatingactivity\left(\%\right)=\left(1-\frac{A_{s}}{A_{b}}\right)*100$$

where $A_{s}$ is the absorbance of the sample, $A_{b}$ is the absorbance of the blank solution without hydrolysates.

#### 2.12.4 Reducing power

The reducing power was measured according to the method of Zou et al. [32] with slight modifications, by mixing 1 mL of SYPHs with 1 mL of 1% (w/v) C_6_FeK_3_N_6_ and 1 mL of 0.2 M sodium phosphate buffer (pH 6.6) and incubated for 20 min at 50 ℃. Thereafter, 1 mL of 10% C_2_HCl_3_O_2_ was added to the mixture and centrifuged at 6250 rpm and 4 ℃ for 10 min. The supernatant (2 mL) was mixed with 0.4 mL of 0.1% (w/v) FeCl_3_ and 2 mL DW, incubated for 10 min at room temperature of 25 ℃, and the absorbance was measured at 700 nm from the multi-mode microplate reader. Better reducing power of hydrolysates was indicated by higher absorbance values and DW was used as the blank.

### 2.13 Statistical analysis

Data were analyzed by analysis of variance (ANOVA) in triplicates from SigmaPlot 12.0 (Systat Software Inc., CA, USA) and Statistix 9.0 (Analytical Software Ltd., Tallahassee, FL, USA), and presented as mean ± standard error of measurement (SEM). at a significance level of *p* < 0.05. Multivariate data analysis of principal component analysis (PCA) and hierarchical cluster analysis (HCA) were performed from SPSS 23.0 (SPSS Inc., Chicago, USA) and OriginPro 9.8.0.200 (OriginLab Corp., Massachusetts, USA).

## 3 Results and discussion

### 3.1 Discharge characteristics of DBD plasma system and physicochemical properties of PFW

**Fig. 2:**
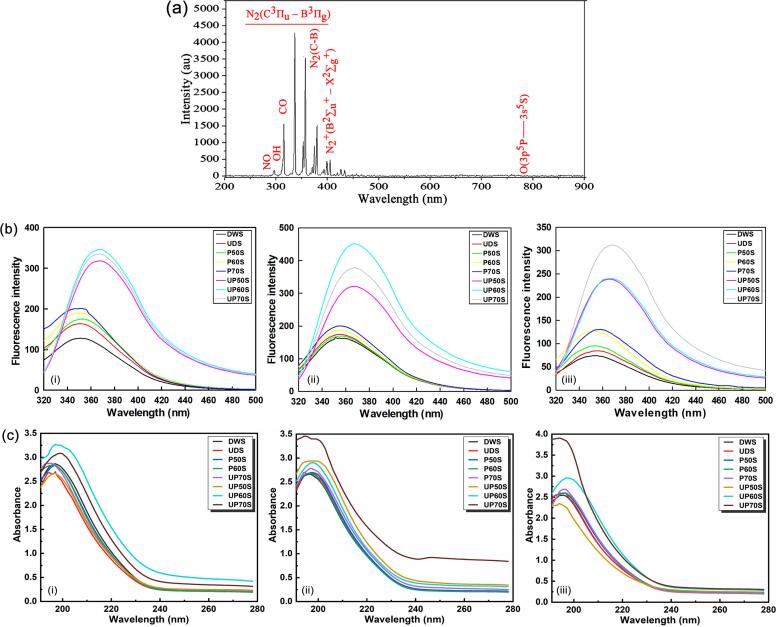
(a) Discharge characteristics of DBD plasma system (c) changes in intrinsic fluorescence and (d) changes in UV–Vis absorption spectra of SYPHs during treatment: (i) CAH (ii) CCH (iii) CPH.

The DBD plasma system utilized air as the processing gas and this ensured an ample supply of oxygen and nitrogen. Analysis of plasma excited species during the generation of PFW could provide insights on PFW-protein interactions in the fish substrate and the mechanism for inducing conformational changes in protein structure. As shown in Fig. 2a, distinct peaks were obtained mainly in the near-UV region compared with the less obviously strong peaks in the near-infrared and visible regions. According to the comparison with the NIST Atomic Spectra Database [26], the emission spectrum was dominated by excited species of atomic nitrogen from the second positive system of N_2_(C-B) at approximately 338, 354, 370, 382, and 405 nm, and the first negative system of N_2_^+^ (B-X) at approximately 357, 395, and 434 nm, which were similar to the report by Misra et al. [33]. Additionally, NO emission, OH peak and optical transition of CO were also identified at 297, 300 and 313 nm, respectively, while very low-intensity peaks around the 780 nm region likely related to the optical transitions of O atoms in air plasma and linked with the quenching of O(^3^P) and O(^5^P) were also observed. The results suggested that DBD plasma was a significant source of chemically reactive species, which were further converted to other RONS in the gas–liquid interface.

Further interactions were observed between radicals and the active ions in the liquid generated various acidic species like nitric acid (HNO_3_), nitrous acid (HNO_2_), and peroxynitrous acid (ONOOH) [18], [34], [35], [36], [37], which was thus responsible for increased acidity of PFW to a pH of 2.60 at 70 V from initial DW pH of 6.67 (Table 1b). Similar results by Bai et al. [38] also indicated increased acidity from 6.71 to 2.84 with increasing activation time and power when DW was exposed to cold plasma. For the temperature, DW had an initial value of 25.30 ℃, which increased rapidly to 44.70 ℃ after 50 V and 49.80 ℃ after 70 V. The concentration of long half-life species of H_2_O_2_ and O_3_ also increased with increasing voltage and reached 2.077 µM and 5.05 mg/L, respectively after 70 V, from initial DW values of 0.0024 µM and 0.07 mg/L. The half-life of H_2_O_2_ has been reported to range from 8 h to 20 days [10]. H_2_O_2_ is regarded as an important oxidant especially in acidic environments to cause the oxidation of proteins and membrane lipids, and a similar increasing trend with increasing activation voltage was reported for DW exposed to air plasma [39].

In order to determine the degree of electron activity, strength and concentration of oxidizers and show evidence of the nature of active ions generated and accumulated in PFW, the ORP and ECT were also measured and the values increased steadily to 598.43 mV and 2.7275 mS/cm, respectively after 70 V from initial DW values of 304.35 mV and 0.0094 mS/cm. The results were attributed to the accumulation of numerous active RONS during exposure of DW to plasma discharge and similar results were reported in the literature [35], [39], [40].

### 3.2 Protein content of samples

Samples presented initial protein content of 210.74 g/kg, which was similar to other studies and is common for aquaculture species [30], [41]. Maximum reductions of 18.33 and 32.84% were observed following individual and combination treatments, respectively (Table 1c). The results might be related to the oxidative breakdown of peptide chains and hydrogen bonds of small yellow croaker proteins, which can alter protein secondary or tertiary structure to influence amino acid content and digestibility [41], [42]. Proteins are liable to quantitative and qualitative changes during processing, and the oxidation of proteins may have been enhanced by increased enzymatic breakdown due to cell membrane destruction of ultrasonic cavitation and the actions of RONS [43], [44]. Reactive species can easily reach cell organelles and phospholipids in the cell membranes to cause oxidation and damage to cell membranes [12], [45], [46], [47]. In related studies, UST at 20 kHz and 150 – 400 W for 5 to 120 min reduced the protein content of shrimp from 255 to 183 mg/g [40] and promoted the degradation and modification of beef proteins [48], which indicated the hydrolyzation of proteins into peptides and amino acids. Likewise, microwave processing, steaming, roasting and boiling were reported to induce the oxidation of sturgeon fillet proteins, especially aromatic amino acids with a variety of modifications in protein primary structure [42]. Overall, the results of the study suggested the potential of UST and PFW for rupturing the peptide chains and modifying the structural conformation of small yellow croaker proteins, which might further influence enzymatic hydrolysis and the characteristic of the hydrolysates produced.

### 3.3 Changes in intrinsic fluorescence

Fluorescence spectroscopy was applied for investigating the environment of aromatic amino acid residues like tyrosine (Tyr), phenylalanine (Phe) and tryptophan (Trp), which generate endogenous fluorescence at certain excitation wavelengths, and for providing information on the variations in the tertiary structure of SYPHs [14]. The fluorescence spectra of SYPHs displayed similar patterns irrespective of the enzyme type with increasing peak fluorescence intensity (FI_peak_) following treatments, relative to control (Fig. 2b). The maximum emission wavelengths of DWS were 351, 351, and 352 nm and a redshift occurred to the maximum of 353, 355 and 358 nm following individual treatments and 369, 368 and 368 nm following combination treatments, respectively, for CAH, CCH and CPH. It was reported that cold plasma could induce hydroxylation of Try, Trp, and Phe, and nitration of Trp and Phe to modify amino acids, which was responsible for the structural unfolding of horseradish peroxidase at the same excitation wavelength of 280 nm [35]. The functional groups of amino acids existing and situated near the cation-binding sites of most enzymes are highly reactive to RONS like ONOO^–^, and electron-donating groups like OH^–^ might influence conjugation to change fluorescence intensity [35]. In the current study, DW was enriched with numerous reactive species and was subsequently converted to various RONS at the gas–liquid interface during functionalization by cold plasma. Also, ultrasound-induced cavitation and pyrolysis reactions may have disrupted hydrophobic interactions of protein molecules to expose buried Trp residues to the hydrophilic environment. The actions of UST and PFW presented a more flexible protein conformation with increased polarity for the structural unfolding of peptide chains, thereby increasing fluorescence intensity [22], [49], which was in agreement with the results for protein analysis. The effects were more pronounced with Chymotrypsin-hydrolysed samples, and the results in the current study were consistent with the findings of Zou et al. [31], where UST increased the fluorescence intensity of chicken plasma protein to a maximum value of 332 nm and partly unfolded the tertiary structure, which was attributed to the fluid mixing of plasma proteins from strong shear forces induced by cavitation, but prolonged treatment for up to 30 min led to over processing and reduction in fluorescence intensity.

### 3.4 Changes in UV–Vis spectra

The UV–Vis spectra of SYPHs were also obtained for monitoring the structural modifications and protein conformation changes, and are presented in Fig. 2c. Data showed peak wavelengths from 190 to 205 nm, which were associated with peptide bonds cleavage and amino acid side chains of typical proteins of Trp, Phe, Tyr in SYPHs [50]. Treatments increased the peak absorbance intensity (AI_peak_) in comparison with DWS, especially Chymotrypsin-hydrolysed samples, possibly due to higher release of soluble proteins and modifications of peptide bonds and free amino acids [1], [35]. The maximum absorbance wavelengths (λ_maxab_) of DWS were 193, 195, and 194 nm and redshifts occurred to the maximum of 197, 198 and 197 nm following individual treatments and 200, 201 and 197 nm following combination treatments, respectively, for CAH, CCH and CPH. The absorption of proteins in the range of 180 – 230 nm arose primarily from the peptide backbone, and to some extent also on the microenvironment and polarity of surrounding amino acids residue groups [14], [50]. Amide groups of peptides exhibited absorption bands near 190 nm that was almost entirely due to π → π ∗ transitions in the peptide bonds, while amino acids side chains of Trp, Phe, Tyr, His, Cys, Met and Arg contributed to the absorbance at 205 nm in descending order [50], [51]. The changes in AI_peak_ and λ_maxab_ also reflected conformational changes due to the exposure of hydrophobic groups, and to a great extent structural unfolding of SYPHs under the oxidative actions of UST and PFW in a similar manner as the change in intrinsic fluorescence intensity. Increases in AI_peak_ and similar shifts in λ_maxab_ were reported for tropomyosin and myofibrillar proteins from king prawn [14], [49] and horseradish peroxidase [20] after exposure to cold plasma, and for tilapia by-products after pressure-assisted enzymatic hydrolysis [1].

### 3.5 Changes in microstructure

**Fig. 3:**
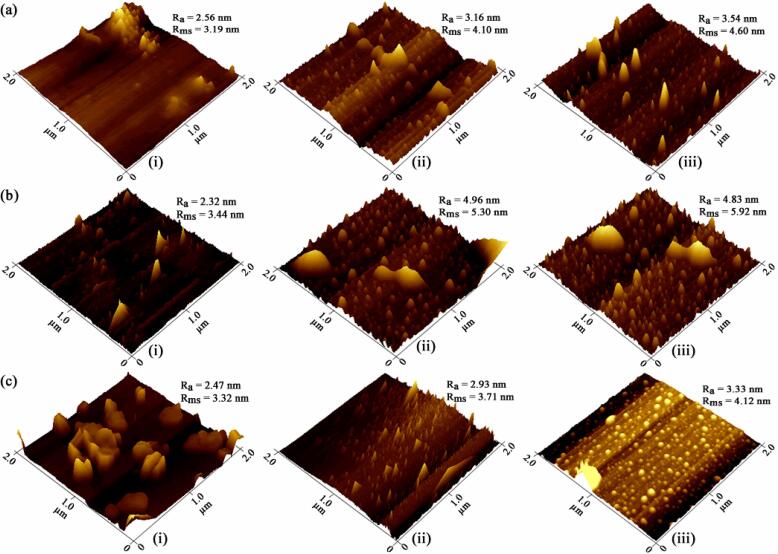
Microstructure images of SYPHs during treatment (a) CAH (b) CCH (c) CPH: (i) DWS (ii) after individual treatments, and (iii) combination treatments.

The microstructure of SYPHs acquired by AFM (Fig. 3) indicated large aggregated structural morphology before treatment and disordered structures with several microparticles and loosed granular structure after treatment, which was similar to the findings of Wang et al. [52]. The average roughnesses (R_a_) of DWS were 2.56, 2.32 and 2.47 nm, and the values increased to 3.16, 4.96 and 2.93 nm following individual treatments and to 3.54, 4.83 and 3.33 nm following combination treatments, respectively, for CAH, CCH and CPH. The root means square roughness (R_ms_) increased to 4.10, 5.30 and 3.71 nm following individual treatments, and to 4.60, 5.92 and 4.12 nm following combination treatments, from DWS values of 3.19, 3.44 and 3.32 nm, respectively, for CAH, CCH and CPH. The oxidative actions of chemically active species like NO∙, O_2_, ∙OH and HOO∙ in PFW and localized high temperature and pressure from cavitation-induced shock waves might disrupt protein aggregation. These actions can damage molecular micropores to increase protein surface roughness with an increased number of smaller sized particles. The surface roughness of proteins typically portrays the overall surface micromorphology, which is vital to enhancing the efficiency of enzymatic hydrolysis, and the current results were similar to the findings of Tian et al. [53] and Wang et al. [52]. Tian et al. [53] reported increased roughness of the surfaces of soy protein hydrolysates due to UST-induced structural disruption of tightly-formed peptide aggregates to smaller fragments with reduced height and diameter. On the other hand, Wang et al. [52] showed that dual-frequency slit UST significantly increased R_a_ and R_ms_ of corn gluten meal to values of 16.29 and 22.32 nm from control values of 2.68 and 6.91 nm, respectively, and suggested a specific correlation between enzymatic substrate characteristics and surface roughness. The significantly higher values observed when compared with the current study might be related to variations in substrates and processing conditions. Overall, the microstructure analysis indicated the significant effects of UST and PFW for increasing surface roughness and reducing the particle size of SYPHs through the structural disruption of peptide aggregation.

### 3.6 Changes in PSD and particle size

**Fig. 4:**
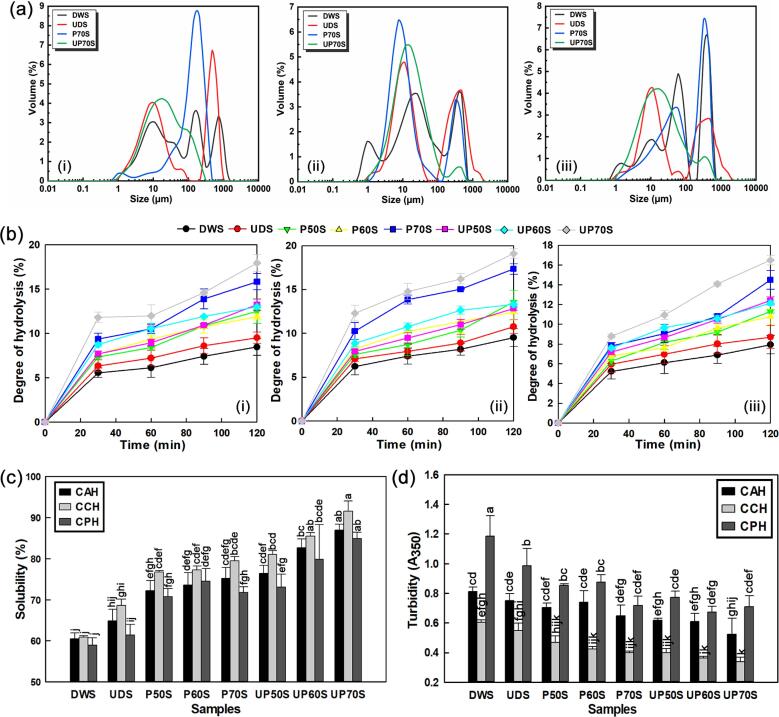
Effects of treatment on (a) particle size distribution and (b) degree of hydrolysis: (i) CAH (ii) CCH (iii) CPH (c) solubility and (d) turbidity of SYPHs.

The PSD data showed that SYPHs initially presented a polymodal distribution and a bimodal distribution after treatments with peaks shifting to positions for smaller particle sizes (Fig. 4a). Treatments increased the SSA and decreased the D_43_, suggesting enhanced adsorption capacity and the promotion of electrostatic repulsion, respectively [21]. The SSA increased to 0.589, 0.649 and 0.437 m^2^/g after individual treatments and 0.644, 0.910 and 0.502 m^2^/g after combination treatments, from DWS values of 0.502, 0.592 and 0.326 m^2^/g, respectively for CAH, CCH and CPH. The D_43_ decreased to 159.57, 91.28 and 178.36 µm after individual treatments and 43.36, 38.74 and 56.14 µm after combination treatments, from DWS values of 181.38, 123.18 and 187.43 µm, respectively, for CAH, CCH and CPH. Particle size influences protein conformational changes, and Chymotrypsin-hydrolysed samples exhibited larger SSA and smaller particle sizes, indicating increased susceptibility compared with other enzymes.

The acidic nature and RONS from PFW, and the shear stress from UST might damage intermolecular hydrophobic and electrostatic interactions between protein molecules to increase molecular collision, resulting in more internal pores [31], [52]. Increased collision and number of internal pores resulted in the collapse of large insoluble protein aggregates and created new surfaces with reduced particle size and increased free surface area. This suggested an enhanced reaction surface for improving enzyme-protein interactions for structural unfolding during hydrolysis [24], [53]. The results were in agreement with the findings of microstructure analysis, and similar effects were reported for chicken breast myofibrillar proteins, attributed to the opening of the protein chains by ultrasound to induce 3-dimensional structural modification for increasing SSA [21]. Previous studies have reported reductions in particle size of king prawn tropomyosin, golden threadfin bream hydrolysates and small yellow croaker myofibrillar proteins from cold plasma and ultrasound treatments [5], [49], [52].

### 3.7 Changes in DoH

The different enzyme types presented similar hydrolysis curves (Fig. 4b), and the DoH increased with increasing hydrolysis times in line with a previous study [54]. The DoH of DWS reached 8.47, 9.52 and 7.94%, compared with 15.84, 17.34 and 14.51% after individual treatments, and 17.94, 19.10 and 16.50% after combination treatments, respectively, for CAH, CCH and CPH. This indicated improved efficiency of enzymatic hydrolysis following treatments, attributed to the cavitation-induced mechanical actions of UST and oxidative RONS of PFW. These actions might disrupt the interactions between sequence stretches of amino acids in the native protein, resulting in unfolding and increased surface area to expose more enzyme binding sites for improving the hydrolysis of peptide bonds [55]. This was also in agreement with the findings for spectral readings, microstructure and particle size analysis as discussed previously.

The active sites of enzymes are influenced by their 3-dimensional structures and conformational changes can impact their activity and substrate specificity [1]. Compared with the other enzymes, Chymotrypsin-hydrolysed samples presented significantly higher DoH, indicating variations in the sequence and amino acid composition of peptides between enzymes and higher efficiency in cleaving peptide bonds [56]. The DoH defines the percentage of cleaved peptide bonds, which is highly related to the hydrolytic process yield, and the enzyme-substrate specificity is a determining factor of the biofunctional properties of hydrolysates as it strongly affects the hydrophilic/hydrophobic balance, molecular size and sequence of peptides [54], [55]. Altınelataman et al. [30] previously reported that Chymotrypsin hydrolysates from European seabass and gilthead seabream exhibited more antioxidant peptides when compared with Alcalase, attributed to the low selectivity of Alcalase. In addition, Klompong et al. [56] suggested that alkaline proteases generally exhibit higher activities in comparison with acidic or neutral proteases, and the pH for Chymotrypsin activity in the current study was more alkaline when compared with the other enzymes (Table 1a). Similar increases in DoH were reported for ultrasound pretreated porcine cerebral hydrolysates and protein hydrolysates from ultrasound and microwave pretreated *Labeo rohita* heads [32], [55]. In another study, improvement in DoH of myofibrillar protein hydrolysates from *Nemipterus virgatus* was linked to improved interactions between myofibrillar proteins and enzymes from increased sites accessible to enzymes due to flexible and loose conformation of proteins as a result of combined ultrasound and microwave treatments [5].

### 3.8 Changes in functional properties

#### 3.8.1 Protein solubility and turbidity

Protein solubility and turbidity are common indicators for evaluating the progression of aggregation and functionality of the proteins in food systems, as any change may influence their bioactivity and potential industrial uses [14], [53]. Soluble proteins enhance interfacial properties and ensure homogenous dispersion of molecules in a colloidal system and are required in various functional food applications [57]. Compared with DWS, the solubility of SYPHs increased by 24.39, 30.60, and 26.46% after individual treatments and 43.80, 50.50, and 44.04% after combination treatments, for CAH, CCH and CPH, respectively (Fig. 4c). In contrast, the turbidity decreased by 19.75, 33.72, and 39.63% after individual treatments and 35.43, 43.69, and 43.17% after combination treatments, for CAH, CCH and CPH, respectively. Overall, treated samples were more soluble and less turbid and Chymotrypsin-hydrolysed samples presented the highest solubility and lowest turbidity when compared with the other enzymes. The results might be related to the degradation of large insoluble protein aggregates to smaller peptides with high solubility, which was in agreement with the findings of microstructure and particle size distribution.

During hydrolysis, exposed hydrophobic areas from unfolded proteins have more possibilities for interactions compared with intact protein and could bind to hydrophobic areas of other molecules to form aggregates [58]. Such aggregation could result in larger aggregating peptides with low solubility and high turbidity, or smaller non-aggregating peptides with more polar residues and the capacity of forming hydrogen bonds with water to enhance solubility and decrease turbidity [53], [56]. Similar findings were reported during partial alteration of the conformation of porcine cerebral and soy protein hydrolysate peptides [32], [53]. Non-covalent and electrostatic interactions were affected, which loosened protein tissue to enhance solubility in comparison with untreated samples. Pressure-assisted enzymatic hydrolysis also led to pronounced increases in the solubility of tilapia by-product protein hydrolysates, but increases in turbidity and corresponding decreases in the solubility of king prawn myofibrillar proteins were reported during cold plasma treatment [1], [14].

#### 3.8.2 Emulsifying properties

**Fig. 5:**
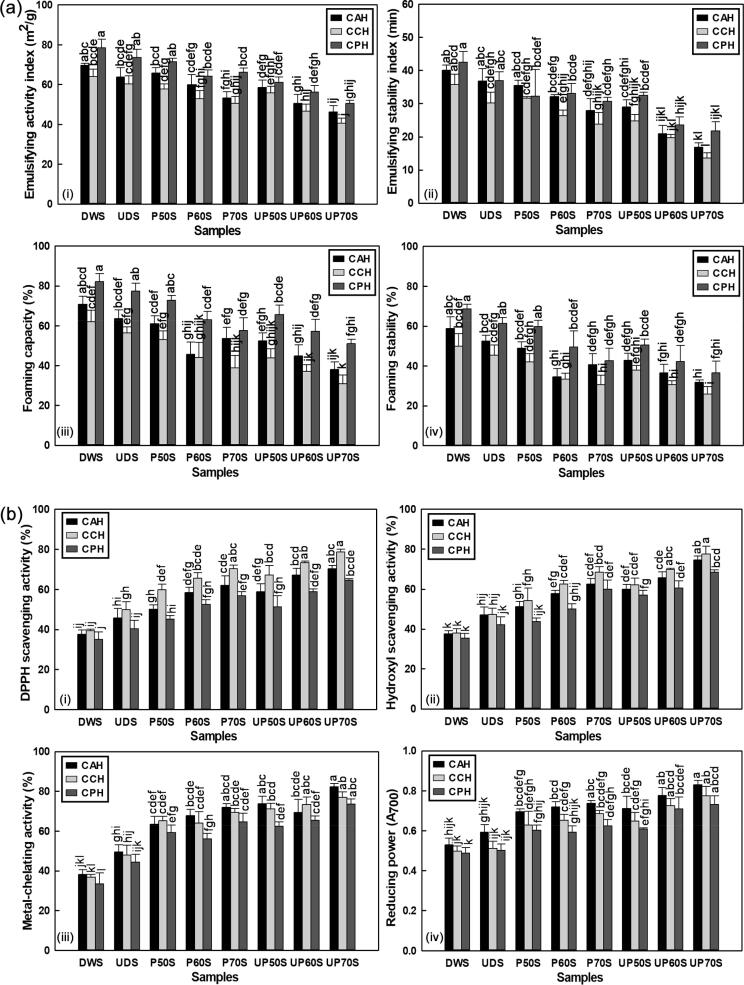
Effects of treatment on (a) functional properties: (i) emulsifying activity index (ii) emulsifying stability index (iii) foaming capacity (iv) foaming stability and (b) antioxidant properties: (i) DPPH radical scavenging activity (ii) hydroxyl radical scavenging activity (iii) metal-chelating activity (iv) reducing power of SYPHs.

The emulsifying properties of proteins, reflected from the EAI and ESI is often used as a reliable indication of enzymatic modification and their effectiveness as potential emulsifying agents in food systems [32], [59]. Relative to DWS, the EAI of SYPHs was significantly reduced by 23.47, 21.12 and 18.11% after individual treatments and 33.66, 36.57 and 35.52% after combination treatments, while the ESI decreased by 30.32, 33.20 and 27.86% after individual treatments and 57.90, 62.06 and 48.60% after combination treatments, respectively, for CAH, CCH and CPH (Fig. 5a(i-ii)). Control samples displayed better emulsifying properties in comparison with treated samples, attributed to the decrease in peptide sizes, leading to enhanced solubility and reduced turbidity of SYPHs. The effects were less pronounced with Papain-hydrolysed samples and suggested varying charges of the resulting peptides, which might be linked to the enzyme-substrate specificity observed during hydrolysis.

Emulsifying properties might be positively correlated with turbidity, and the formation of small particle-sized peptides during hydrolysis were linked with weak interfacial activities that reduced emulsifying properties [1], [32]. Emulsions are produced from the adsorption of peptides on the surfaces of newly formed oil droplets during homogenization and the creation of protective membranes that prevent coalescence of the oil droplets [56]. Hydrolysates are typically surface-active materials and tend to promote oil in water emulsion due to associated charges from hydrophobic and hydrophilic groups [56], [57]. Thus, hydrolysates with small peptide sizes display poor emulsifying properties since small peptides exhibit low efficiency in reducing the oil–water interface tension, from their inability to reorient and unfold at the interface for stabilizing emulsions like large-sized peptides [32]. The results were consistent with the findings of Zou et al. [32], who reported reductions in EAI and ESI of porcine cerebral hydrolysate peptides following ultrasound pretreatment, and those of Hemker et al. [1], who showed that EAI and ESI of tilapia by-product protein hydrolysates tended to reduce with increasing pressure and holding time during pressure-assisted enzymatic hydrolysis. In contrast, significant increases in EAI and ESI were reported for natural actomyosin from king prawns, attributed to the increase in particle sizes of proteins during cold plasma treatment [59].

#### 3.8.3 Foaming properties

The ability of SYPHs to create foams was significantly reduced following treatments in comparison with DWS (Fig. 5a(iii-iv)). Particularly, FCY was reduced by 35.66, 37.22 and 29.87% after individual treatments and 46.43, 50.27 and 37.89% after combination treatments, while FSY was reduced by 41.12, 38.44 and 37.89% after individual treatments and 45.89, 48.18 and 46.78% after combination treatments, respectively for CAH, CCH and CPH. The results also showed that Papain-hydrolysed samples displayed better foaming properties, closely followed by Alcalase-hydrolysed samples. The organoleptic properties of foam products are primarily influenced by the diameter of air bubbles in foams. Foam formation is governed by the penetration, transportation and reorganization of molecules at the air–water interface. It is dependent upon colloidal interface adsorption of a layer of the denatured protein surface that is attached to the liquid with air entrapment to generate bubbles [14], [57]. Conformational changes and amino acid hydroxylation can influence protein chain flexibility from adsorption at the air–water interface and impact the elasticity of the adsorbed layer [14]. Thus, the nitration and hydroxylation of amino acids, which increased fluorescence intensity might be accompanied by weak interfacial adsorption, resulting in bubble collapse and thereby reducing the foaming properties of SYPHs. Similar results were reported for porcine cerebral hydrolysate where foam expansion and foam stability were significantly reduced, attributed to low molecular weight peptides and increased solubility of the hydrolysate following UST [32], while cold plasma treatment did not produce significant differences in the foaming properties of myofibrillar proteins from king prawn [14]. Furthermore, large aggregating peptides were reported to positively correlate with foam stability of hydrolysates as microscopic or smaller peptides did not possess the needed strength to maintain stable foams [56]. Microstructure analysis and particle size evaluation suggested depolymerisation of large aggregates to smaller fragments with high solubility after treatment, and the coinciding of the least foaming properties with the highest solubility of SYPHs.

### 3.9 Changes in antioxidant activities

#### 3.9.1 DPPH and hydroxyl radical scavenging activity

The DPPH and hydroxl radical scavenging activities of SYPHs are presented in Fig. 5b(i)-(ii). For DPPH radical, the values were 37.58, 39.37, and 34.92% for DWS, which increased to 61.91, 70.25, and 56.71% after individual treatments, and to 70.31, 78.68, and 64.54% after combination treatments, respectively for CAH, CCH and CPH. For hydroxyl radical, the values were 37.45, 37.98, and 35.40% for DWS, which increased to 62.51, 68.36, and 59.85% after individual treatments, and to 74.59, 77.51, and 68.44% after combined treatments, respectively for CAH, CCH and CPH. Antioxidant activity is an important index for measuring protein bioactivity and represents the type and amount of peptides generated during hydrolysis [53]. DPPH radical scavenging activity expresses the ability to transform active free radicals to more stable products, while the elimination of the highly reactive oxygen species from the hydroxyl radical is regarded as an effective defence mechanism against numerous psychological disorders [5], [32]. It was possible that increased peptide concentration during hydrolysis aided by UST and PFW generated more radical scavenging fragments, which might contribute to stabilizing the active DPPH and scavenging hydroxyl radicals, and the findings were consistent with the report of Zou et al. [32].

Besides, the results indicated that Chymotrypsin-hydrolysed samples presented higher DPPH and hydroxyl scavenging activities and could more effectively act as radical donors in comparison with other enzymes, which might be related to the release of more antioxidant peptides that was earlier reported for this enzyme during hydrolysis. The antioxidant activity of protein hydrolysates is influenced by the hydrolysis conditions and enzyme specificity, as a variety of free amino acid sequences and smaller peptides are generated, and these variations may be responsible for the differing radical scavenging activities from the different enzymes [29], [56]. Similar enzyme-substrate specific variations in DPPH and hydroxyl scavenging activity were reported in the literature. For instance, enhanced DPPH and hydroxyl radical scavenging activities were reported for Alcalase-hydrolyzed golden threadfin bream and by-products of striped weakfish protein hydrolysates, respectively, in comparison with Pepsin, Proteinase and Protamex [5], [29], enhanced DPPH radical scavenging activity was also reported to correlate with increasing DoH of Alcalase-hydrolyzed samples during combined ultrasound-microwave treatment, but improvements in hydroxyl radical scavenging activity was not associated with a distinct pattern in DoH [5], while improvement in DoH was reported to typically associate with exposed hydrogen donors from shorter peptide fragments with fast-molecular-motion for attacking more free radicals, and ultrasound treatment thus caused the breaking up of peptide agglomerates and released small peptides with high antioxidant capacity for increasing the radical scavenging activity of soy protein hydrolysates [53]. In addition, improved DPPH radical scavenging activity was reported for tilapia by-product protein hydrolysates during pressure-assisted enzymatic hydrolysis, and this was attributed to the release of low molecular weight peptides by pressure treatment (100 – 400 MPa) in comparison with atmospheric pressure during hydrolysis [1].

#### 3.9.2 Metal-chelating activity and reducing power

The antioxidant activity of SYPHs was also shown in Fig. 5b(iii-iv) in terms of their metal-chelating activity and reducing power. The metal-chelating activity increased to 72.01, 69.29, and 64.64% after individual treatments and to 82.14, 76.95, and 73.45% after combination treatments, from 38.10, 36.78, and 33.48% for DWS, respectively for CAH, CCH and CPH. The reducing power increased to 0.737, 0.687, and 0.625 after individual treatments and to 0.830, 0.775, and 0.732 after combination treatments, from 0.530, 0.497, and 0.487 for DWS, respectively for CAH, CCH and CPH. Alcalase-hydrolyzed samples exhibited superior chelating activity and reducing power in comparison with Chymotrypsin and Papain, further confirming enzyme-substrate specificity, which was consistent with other studies [28], [29], [54]. Khantaphant et al. [28] reported higher metal-chelating activity for brownstripe red snapper hydrolysates prepared from Flavourzyme in comparison with Alcalase during a two-step hydrolytic process using pyloric caeca protease. Flavourzyme, which is a mixture of *exo*-and *endo*-peptidase enzymes was seen as capable of producing both peptides and amino acids to enhance metal-chelating activity in comparison with the *endo*-peptidase Alcalase, which produced only peptides with broad specificity, especially the uncharged residues. Likewise, peptides obtained with Protamex exhibited a higher capacity to chelate Fe^3+^ in comparison with Alcalase during the production of hydrolysates from industrial by-products of striped weakfish [29], while silver carp fin hydrolysates produced by Alcalase and trypsin presented comparatively higher Fe^3+^ chelating activity when compared with Neutrase and Papain [54], and the variations were attributed to the differences in enzyme cleavage positions, exposed side chains of peptide bonds and varying amino acid sequences, which is governed by the specificity of proteases towards protein peptide bonds.

Transition metals like Cu^2+^ and Fe^2+^ influence the rate of autoxidation in fish products by donating electrons to form alkoxyl radicals, thus, chelation of the oxidized form of these ions by antioxidant peptides would reduce their redox potential and retard volatile compound formation [32], [54], [57]. Enhanced metal-chelating activity might be attributed to improved Fe^2+^ binding due to increased amino and carboxylic (COO^–^) group branches of acidic and basic amino acids present in peptide terminals, and higher cleavage of peptide bonds during hydrolysis aided by UST and PFW [54], [56].

Furthermore, reducing power is correlated directly with antioxidant activity as it evaluates electron-donating capacity, and the improved reducing power indicated the strong ability of SYPHs to interact with and donate electrons to ferric ions, which was ascribed to the improved cleavage of protein molecules from enhanced hydrolysis facilitated by UST and PFW [1], [32]. In related studies, ultrasound treated porcine cerebral hydrolysates were found to exhibit stronger reducing power and chelating activity when compared with untreated samples, while reducing power was not influenced by hydrolysis at atmospheric conditions, but showed significant increases under pressure-assisted hydrolysis [1], [32].

### 3.10 Multivariate data analysis

**Fig. 6:**
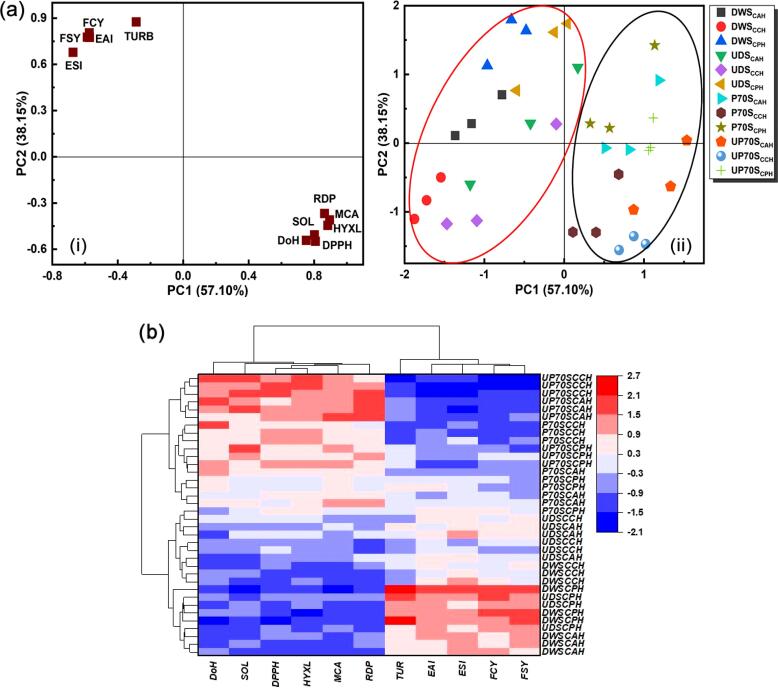
Multivariate data analysis of the properties of SYPHs under different treatment conditions (a) principal component analysis of (i) loading variable plot and (ii) comprehensive score plot (b) heat map combined with dendrogram of hierarchical cluster analysis (EAI: emulsifying activity index, ESI: emulsifying stability index, FCY: foaming capacity, FSY: foaming stability, TURB: turbidity, DoH: degree of hydrolysis, SOL: solubility, RDP: reducing power, MCA: metal chelating activity, HYXL: hydroxyl radical scavenging activity, DPPH: DPPH radical scavenging activity).

PCA was performed for comparing and discriminating SYPHs from different treatments. Fig. 6a(i) shows that PC1 explained 57.10% of the total variance, and DoH, solubility and antioxidant activities were clustered in the positive PC1, indicating strong dependency of the changes in solubility and antioxidant activities on DoH. Turbidity, emulsifying, and foaming properties were also clustered in the positive PC2, which explained 38.15% of the total variance, and indicated that the variations in emulsifying and foaming properties were strongly related to the changes in turbidity. Together, PC1 and PC2 explained 95.25% of the total variance in the properties of SYPHs with Kaiser-Meyer-Olkin measure of sampling adequacy and chi-square values of 0.927 and 700.56, respectively, suggesting feasibility and sufficiency of the PCA [60], [61]. SYPHs from the different treatments exhibited two distinct clusters: DWS and UDS (red circle), P70S and UP70S UDWS (black circle) (Fig. 6a(ii)), and data clustering together normally suggests high similarity in relation to the studied parameters. The heat map combined with the HCA dendrogram further confirmed the results of the PCA, which also showed two main clusters of DWS and UDS, and P70S and UP70S (Fig. 6b). The properties of SYPHs from the different treatments were clustered from the amalgamation rule using Ward’s linkage method and squared Euclidean distance, with colour lumps indicating values of the corresponding variables. Two main clusters were also identified for the properties of SYPHs, and in the first cluster comprising DoH, solubility and antioxidant properties, DWS and UDS presented low values, while P70S and UP70S presented high values in the second cluster including turbidity, emulsifying and foaming properties. Overall, UP70S_CCH_ presented high DoH, solubility, and antioxidant activities, and low emulsifying and foaming properties, while the DWS group showed low DoH, high turbidity, emulsifying and foaming properties and shared more similarities with the UDS group. The analysis revealed that functional and bioactive properties of SYPHs varied with the treatment methods and the enzyme type played an important role in specifying the differences in properties.

## 4 Conclusions

The combination of PFW with ultrasound treatment (UPFW) disrupted intermolecular hydrophobic and electrostatic interactions between protein molecules to break down peptide chains and reduce the protein content of small yellow croaker. The actions also exposed buried amino acid residues with increased polarity to induce conformational changes and structural unfolding, and increase collision between protein molecules to cause the collapse of large insoluble protein aggregates. This created new surfaces with increased roughness and surface area, and smaller particles for improving enzyme-protein interactions. The improved interactions accelerated the hydrolytic process to produce highly soluble peptides with improved antioxidant properties and reduced foaming and emulsifying properties. The overall functionality of SYPHs was influenced by a range of treatment parameters, conditions of hydrolysis and enzyme types, indicating enzyme-specific susceptibility of small yellow croaker to hydrolysis, and Chymotrypsin-hydrolysed small yellow croaker treated with a combination of UST and PFW generated at 70 V presented better results. Absolute discrimination and differentiation of SYPHs from the enzyme types and treatments were achieved through multivariate analysis of PCA and HCA. The findings from the current study should be applicable to the potential utilization of peptides produced during enzymatic hydrolysis of small yellow croaker, therefore, optimization studies are necessary for obtaining desirable characteristics of SYPHs for specific industrial applications.

Compliance with Ethical Standards.

**Funding**: The authors are grateful to the National Natural Science Foundation of China (31972205) for its support. This research was also supported by the International S&T Cooperation Projects of Guangdong Province (2020A0505100007), Guangdong Basic and Applied Basic Research Foundation (2021A1515010644), the Contemporary International Collaborative Research Centre of Guangdong Province on Food Innovative Processing and Intelligent Control (2019A050519001), the Guangzhou Key Laboratory for Intelligent Sensing and Quality Control of Agricultural Products (202102100009) and the Common Technical Innovation Team of Guangdong Province on Preservation and Logistics of Agricultural Products (2021KJ145).In addition, Okon Johnson Esua is in receipt of a PhD scholarship (2018GXZ013452) from the China Scholarship Council.

## Ethical approval

This article does not contain any studies with human participants performed by any of the authors.

## CRediT authorship contribution statement

**Okon Johnson Esua:** Writing – original draft, Formal analysis, Investigation. **Da-Wen Sun:** Supervision, Funding acquisition, Resources, Writing – review & editing. **Jun-Hu Cheng:** Validation, Funding acquisition, Resources. **Huifen Wang:** Investigation. **Mingchun Lv:** Investigation.

## Declaration of Competing Interest

The author declares that he/she has no conflict of interest.

## References

[1] Hemker A.K., Nguyen L.T., Karwe M., Salvi D. (2020). Effects of pressure-assisted enzymatic hydrolysis on functional and bioactive properties of tilapia (Oreochromis niloticus) by-product protein hydrolysates. LWT – Food Sci Technol..

[2] Gao M.-T., Hirata M., Toorisaka E., Hano T. (2006). Acid-hydrolysis of fish wastes for lactic acid fermentation. Bioresour. Technol..

[3] Bashir K.M.I., Sohn J.H., Kim J.-S., Choi J.-S. (2020). Identification and characterization of novel antioxidant peptides from mackerel (Scomber japonicus) muscle protein hydrolysates. Food Chem..

[4] Melgosa R., Trigueros E., Sanz M.T., Cardeira M., Rodrigues L., Fernandez N., Matias A.A., Bronze M.R., Marques M., Paiva A., Simoes P. (2020). Supercritical CO2 and subcritical water technologies for the production of bioactive extracts from sardine (Sardina pilchardus) waste. J. Supercrit. Fluids.

[5] Li Z., Wang J., Zheng B., Guo Z. (2020). Impact of combined ultrasound-microwave treatment on structural and functional properties of golden threadfin bream (Nemipterus virgatus) myofibrillar proteins and hydrolysates. Ultrason. Sonochem..

[6] Rivero-Pino F., Espejo-Carpio J.F., Guadix E.M. (2020). Bioactive fish hydrolysates resistance to food processing. LWT - Food Sci. Technol..

[7] Ekezie F.-G.C., Cheng J.-H., Sun D.-W. (2017). A review on recent advances in cold plasma technology for the food industry: Current applications and future trends, Trend. Food Sci. Technol..

[8] Han Y.-X., Cheng J.-H., Sun D.-W. (2019). Activities and conformation changes of food enzymes induced by cold plasma: A review. Crit. Rev. Food Sci. Nutr..

[9] Chen Y.-Q., Cheng J.-H., Sun D.-W. (2020). Chemical, physical and physiological quality attributes of fruit and vegetables induced by cold plasma treatment: mechanisms and application advances. Crit. Rev. Food Sci. Nutr..

[10] Esua O.J., Cheng J.-H., Sun D.-W. (2020). Antimicrobial activities of plasma-functionalized liquids against foodborne pathogens on grass carp (Ctenopharyngodon idella). Appl. Microbiol. Biotechnol..

[11] Ali M., Cheng J.-H., Sun D.-W. (2021). Effect of dielectric barrier discharge cold plasma treatments on degradation of anilazine fungicide and quality of tomato (Lycopersicon esculentum Mill) juice. Int. J. Food Sci. Technol..

[12] Esua O.J., Cheng J.-H., Sun D.-W. (2021). Functionalization of water as a nonthermal approach for ensuring safety and quality meat and seafood products. Crit. Rev. Food Sci. Nutr..

[13] Ali M., Cheng J.-H., Sun D.-W. (2021). Effect of plasma activated water and buffer solution on fungicide degradation from tomato (Solanum lycopersicum) fruit. Food Chem..

[14] Ekezie F.-G.C., Cheng J.-H., Sun D.-W. (2019). Effects of atmospheric pressure plasma jet on the conformation and physicochemical properties of myofibrillar proteins from king prawn (Litopenaeus vannamei). Food Chem..

[15] Zhang P., Zhu Z., Sun D.-W. (2018). Using power ultrasound to accelerate food freezing processes: effects on freezing efficiency and food microstructure. Crit. Rev Food Sci. Nutr..

[16] Esua O.J., Chin N.L., Yusof Y.A., Sukor R. (2020). A review on individual and combination technologies of UV-C radiation and ultrasound in postharvest handling of fruits and vegetables. Processes.

[17] Esua O.J., Cheng J.-H., Sun D.-W. (2021). Novel technique for treating grass carp (Ctenopharyngodon idella) by combining plasma functionalized liquids and ultrasound: Effects on bacterial inactivation and quality attributes. Ultrason. Sonochem..

[18] Esua O.J., Cheng J.-H., Sun D.-W. (2021). Optimisation of treatment conditions for reducing Shewanella putrefaciens and Salmonella Typhimurium on grass carp treated by thermoultrasound-assisted plasma functionalized buffer. Ultrason. Sonochem..

[19] Bangar S.P., Esua O.J., Sharma N., Thirumdas R. (2022). Ultrasound-assisted modification of gelation properties of proteins: A review. J. Texture Stud..

[20] Sun Q., Chen Q., Xia X., Kong B., Diao X. (2019). Effects of ultrasound-assisted freezing at different power levels on the structure and thermal stability of common carp (Cyprinus carpio) proteins. Ultrason. Sonochem..

[21] Chen J., Zhang X., Fu M., Chen X., Pius B.A., Xu X. (2021). Ultrasound-assisted covalent reaction of myofibrillar protein: The improvement of functional properties and its potential mechanism. Ultrason. Sonochem..

[22] Li Z., Zheng Y., Sun Q., Wang J., Zheng B., Guo Z. (2021). Structural characteristics and emulsifying properties of myofibrillar protein-dextran conjugates induced by ultrasound Maillard reaction. Ultrason. Sonochem..

[23] Ma B., Wang L., Lou B., Tan P., Xu D., Chen R. (2020). Dietary protein and lipid levels affect the growth performance, intestinal digestive enzyme activities and related genes expression of juvenile small yellow croaker (*Larimichthys polyactis*). Aquac. Rep..

[24] Wang Y.-Y., Rashid M.T., Yan J.-K., Ma H. (2021). Effect of multi-frequency ultrasound thawing on the structure and rheological properties of myofibrillar proteins from small yellow croaker. Ultrason. Sonochem..

[25] Pan Y., Zhang Y., Cheng J.-H., Sun D.-W. (2020). Inactivation of *Listeria monocytogenes* at various growth temperatures by ultrasound pretreatment and cold plasma. LWT - Food Sci. Technol..

[26] NIST (2018).

[27] AOAC (2000).

[28] Khantaphant S., Benjakul S., Kishimura H. (2011). Antioxidative and ACE inhibitory activities of protein hydrolysates from the muscle of brownstripe red snapper prepared using pyloric caeca and commercial proteases. Process Biochem..

[29] Lima K.O., de Quadros C., da Rocha M., de Lacerda J.T.J.G., Juliano M.A., Dias M., Mendes M.A., Prentice C. (2019). Bioactivity and bioaccessibility of protein hydrolysates from industrial byproducts of Stripped weakfish (*Cynoscion guatucupa*), LWT – Food Sci. Technol..

[30] Altınelataman C., Koroleva O., Fedorova T., Torkova A., Lisitskaya K., Tsentalovich M., Kononikhin A., Popov I., Vasina D., Kovalyov L., Celik U. (2019). An in vitro and in silico study on the antioxidant and cell culture-based study on the chemoprotective activities of fish muscle protein hydrolysates obtained from European seabass and gilthead seabream. Food Chem..

[31] Zou Y., Yang H., Li P.P., Zhang M.H., Zhang X.X., Xu W.M., Wang D.Y. (2019). Effect of different time of ultrasound treatment on physicochemical, thermal, and antioxidant properties of chicken plasma protein. Poult. Sci..

[32] Zou Y., Wang W., Li Q., Chen Y., Zheng D., Zou Y., Zhang M., Zhao T., Mao G., Feng W., Wu X., Yang L. (2016). Physicochemical, functional properties and antioxidant activities of porcine cerebral hydrolysate peptides produced by ultrasound processing. Process Biochem..

[33] Misra N.N., Pankaj S.K., Walsh T., O'Regan F., Bourke P., Cullen P.J. (2014). In-package nonthermal plasma degradation of pesticides on fresh produce. J. Hazard. Mater..

[34] Pan Y., Cheng J.-H., Sun D.-W. (2019). Cold plasma-mediated treatments for shelf life extension of fresh produce: A review of recent research developments. Compr. Rev. Food Sci. Food Saf..

[35] Han Y.-X., Cheng J.-H., Sun D.-W. (2019). Changes in activity, structure and morphology of horseradish peroxidase induced by cold plasma. Food Chem..

[36] Jiang Y.-H., Cheng J.-H., Sun D.-W. (2020). Effects of plasma chemistry on the interfacial performance of protein and polysaccharide in emulsion. Trends Food Sci. Technol..

[37] Pan Y.-W., Cheng J.-H., Sun D.-W. (2021). Inhibition of fruit softening by cold plasma treatments: affecting factors and applications. Crit. Rev. Food Sci. Nutr..

[38] Bai Y., Muhammad A.I., Hu Y., Koseki S., Liao X., Chen S., Ye X., Liu D., Ding T. (2020). Inactivation kinetics of *Bacillus cereus* spores by plasma activated water (PAW). Food Res. Int..

[39] Xiang Q., Kang C., Niu L., Zhao D., Li K., Bai Y. (2018). Antibacterial activity and a membrane damage mechanism of plasma-activated water against *Pseudomonas deceptionensis* CM2, LWT-Food. Sci. Technol..

[40] Ali M., Sun D.-W., Cheng J.-H., Esua O.J. (2022). Effects of combined treatment of plasma activated liquid and ultrasound for degradation of chlorothalonil fungicide residues in tomato. Food Chem.

[41] Dong X., Wang J., Raghavan V. (2020). Effects of high-intensity ultrasound processing on the physicochemical and allergenic properties of shrimp. Innov. Food Sci. Emerg. Technol..

[42] Hu L., Ren S., Shen Q., Chen J., Ye X., Ling J. (2017). Proteomic study of the effect of different cooking methods on protein oxidation in fish fillets. RSC Adv..

[43] Abraha B., Admassu H., Mahmud A., Tsighe N., Shui X.-W., Fang Y. (2018). Effect of processing methods on nutritional and physico-chemical composition of fish: a review. MOJ Food Process. Technol..

[44] Esua O.J., Sun D.-W., Cheng J.-H., Wang H., Chen C. (2022). Hybridising plasma functionalized water and ultrasound pretreatment for enzymatic protein hydrolysis of *Larimichthys polyactis*: Parametric screening and optimization. Food Chem..

[45] Pan Y., Cheng J.-H., Lv X., Sun D.-W. (2019). Assessing the inactivation efficiency of Ar/O2 plasma treatment against *Listeria monocytogenes* cells: Sublethal injury and inactivation kinetics. LWT - Food Sci. Technol..

[46] Wu Y., Cheng J.-H., Sun D.-W. (2022). Subcellular damages of *Colletotrichum asianum* and inhibition of mango anthracnose by dielectric barrier discharge plasma. Food Chem..

[47] Zhu H., Han Z., Cheng J.-H., Sun D.-W. (2022). Modification of cellulose from sugarcane (*Saccharum officinarum*) bagasse pulp by cold plasma: Dissolution, structure and surface chemistry analysis. Food Chem.

[48] Kang D.-C., Gao X.-Q., Ge Q.-F., Zhou G.-H., Zhang W.-G. (2017). Effects of ultrasound on the beef structure and water distribution during curing through protein degradation and modification. Ultrason. Sonochem..

[49] Ekezie F.-G.C., Sun D.-W., Cheng J.-H. (2019). Altering the IgE binding capacity of king Prawn (*Litopenaeus Vannamei*) tropomyosin through conformational changes induced by cold argon-plasma jet. Food Chem..

[50] Saraiva M.A. (2020). Interpretation of α-synuclein UV absorption spectra in the peptide bond and the aromatic regions. J. Photochem. Photobiol. B, Biol..

[51] Pereverzev A.Y., Kopysov V.N., Boyarkin O.V. (2018). Peptide bond ultraviolet absorption enables vibrational cold-ion spectroscopy of nonaromatic peptides. J. Phys. Chem. Lett..

[52] Wang Y., Zhang Z., He R., Liu D., Mintah B.K., Dabbour M., Ma H. (2020). Improvement in enzymolysis efficiency and changes in conformational attributes of corn gluten meal by dual-frequency slit ultrasonication action. Ultrason. Sonochem..

[53] Tian R., Feng J., Huang G., Tian B., Zhang Y., Jiang L., Sui X. (2020). Ultrasound driven conformational and physicochemical changes of soy protein hydrolysates. Ultrason. Sonochem..

[54] Zhang L., Shan Y., Hong H., Luo Y., Hong X., Ye W. (2020). Prevention of protein and lipid oxidation in freeze-thawed bighead carp (*Hypophthalmichthys nobilis*) fillets using silver carp (*Hypophthalmichthys molitrix*) fin hydrolysates. LWT-Food Sci. Technol..

[55] Bruno S.F., Kudre T.G., Bhaskar N. (2019). Effects of different pretreatments and proteases on recovery, umami taste compound contents and antioxidant potentials of *Labeo rohita* head protein hydrolysates. J. Food Sci. Technol..

[56] Klompong V., Benjakul S., Kantachote D., Shahidi F. (2007). Antioxidative activity and functional properties of protein hydrolysate of yellow stripe trevally (*Selaroides leptolepis*) as influenced by the degree of hydrolysis and enzyme type. Food Chem..

[57] Sripokar P., Benjakul S., Klomklao S. (2019). Antioxidant and functional properties of protein hydrolysates obtained from starry triggerfish muscle using trypsin from albacore tuna liver, Biocatal. Agric. Biotechnol..

[58] Creusot N., Gruppen H. (2007). Enzyme-induced aggregation and gelation of proteins. Biotechnol. Adv..

[59] Ekezie F.-G.C., Cheng J.-H., Sun D.-W. (2018). Effects of mild oxidative and structural modifications induced by argon-plasma on physicochemical properties of actomyosin from king prawn (*Litopenaeus Vannamei*). J. Agric. Food Chem..

[60] Tian Y., Chen Z., Zhu Z., Sun D.-W. (2020). Effects of tissue pre-degassing followed by ultrasound-assisted freezing on freezing efficiency and quality attributes of radishes. Ultrason. Sonochem..

[61] Tian Y., Zhang P., Zhu Z., Sun D.-W. (2020). Development of a single/dual-frequency orthogonal ultrasound-assisted rapid freezing technique and its effects on quality attributes of frozen potatoes. J. Food Eng..

